# Recurrent Bacteremia in the Setting of Pseudomonas Endocarditis of the Tricuspid Valve and Indwelling Medical Devices

**DOI:** 10.7759/cureus.76368

**Published:** 2024-12-25

**Authors:** Ethan Miller, Hera Jamal, Parita Patel

**Affiliations:** 1 Internal Medicine, Cooper Medical School of Rowan University, Camden, USA; 2 Internal Medicine, Cooper University Hospital, Camden, USA

**Keywords:** bacteremia, biofilm formation, indwelling medical devices, infective endocarditis, pseudomonas aeruginosa, recurrent infection, tricuspid valve endocarditis

## Abstract

This case report presents a complex and challenging scenario of recurrent *Pseudomonas aeruginosa* (*P. aeruginosa*) bacteremia and tricuspid valve endocarditis in a 77-year-old male patient with multiple comorbidities and indwelling medical devices. The patient’s medical history was significant for T4 paraplegia, neurogenic bladder requiring a chronic indwelling suprapubic catheter, heart block status post-permanent pacemaker placement, type 2 diabetes mellitus, chronic kidney disease, and chronic sacral wounds. The case highlights the difficulties in managing antibiotic-resistant *P. aeruginosa* infections, particularly in patients with implantable devices and chronic wounds. The patient’s clinical course was marked by multiple hospital admissions, each time presenting with fever, confusion, and positive blood cultures for *P. aeruginosa*. Despite aggressive antibiotic treatment and interventions such as pacemaker replacement and tricuspid valve debulking, the patient’s condition continued to deteriorate. The recurring nature of the infection, despite therapeutic interventions, underscores the risk of bacterial seeding of indwelling medical devices and the challenges posed by antibiotic resistance. This case also draws attention to the significance of *P. aeruginosa* as a causative agent of severe nosocomial infections, particularly in immunocompromised individuals, and its growing resistance to antibiotics through mechanisms such as biofilm formation. Ultimately, the patient developed septic shock and transitioned to comfort care due to treatment failure, highlighting the difficult clinical decisions required in the face of chronic infections refractory to repeated interventions. This case serves as a reminder of the need for continued vigilance and innovative strategies, such as multifunctional antibacterial-coated devices, in preventing and managing device-associated infections. This is particularly important in the context of increasing antibiotic resistance and the complications associated with biofilm formation.

## Introduction

*Pseudomonas aeruginosa* (*P. aeruginosa*) is an opportunistic gram-negative bacterium that causes severe nosocomial infections in immunocompromised individuals, which can often be fatal. In the United States alone, *P. aeruginosa *is estimated to be responsible for more than 50,000 healthcare-associated infections annually and approximately 440 deaths [1]. Unfortunately, *P. aeruginosa *exhibits growing antibiotic resistance, making these infections increasingly difficult to treat. *P. aeruginosa* often gains antibiotic resistance through biofilm formation, particularly on implantable devices such as pacemakers and indwelling catheters. Biofilms are structured communities of bacteria encased in an extracellular polymeric substance matrix composed of polysaccharides and proteins. This matrix enhances bacterial survival and resistance to antibiotics and the host immune response through mechanisms such as creating a physical barrier, facilitating horizontal gene transfer of antibiotic resistance genes, and upregulating efflux pumps and antibiotic-degrading enzymes [2]. Infective endocarditis frequently occurs in these populations due to chronic bacteremia. Intravenous drug use is the most common cause of *P. aeruginosa* endocarditis, which typically infects the tricuspid valve (TV) [1]. In the absence of intravenous drug use, *P. aeruginosa* endocarditis is often associated with healthcare interventions such as catheterization or implantable devices and underlying comorbidities such as diabetes mellitus. This case highlights the treatment failure of recurrent *P. aeruginosa* infection in a patient with such predisposing factors and multiple potential sources of recurrent infection in the absence of intravenous drug use.

## Case presentation

**Figure 1:**
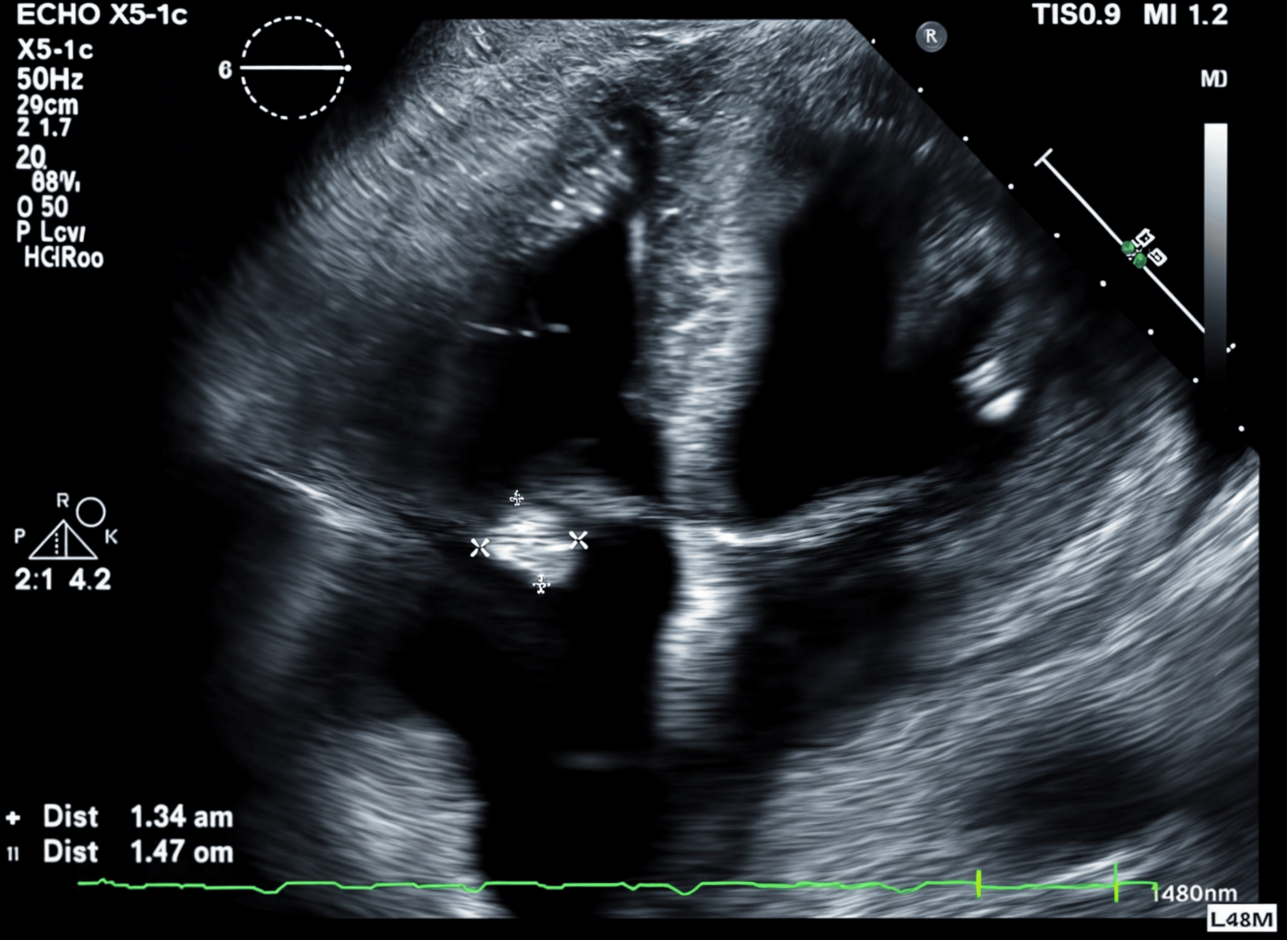
1.3 x 1.5 cm calcified mobile echodensity (indicated by surrounding arrows) on the tip of the anterior leaflet of the tricuspid valve

A 77-year-old male with a past medical history of T4 paraplegia secondary to arteriovenous malformation, neurogenic bladder with a chronic indwelling suprapubic catheter, second-degree heart block status post-permanent pacemaker (PPM) placement, type 2 diabetes mellitus, chronic kidney disease, morbid obesity, and chronic sacral wounds presented with fever and confusion. Urine and blood cultures were positive for *P. aeruginosa*, alongside elevated white blood cell count, lactic acid, C-reactive protein, and erythrocyte sedimentation rate. Due to concerns of infected PPM wires, the device was replaced with a leadless Micra pacemaker to address one of the potential sources of bacterial seeding and reduce the risk of re-infection. The initial infection was treated with meropenem and suprapubic catheter exchange. Meropenem was chosen based on hospital-specific antibiogram data indicating 92% susceptibility of *P. aeruginosa* isolates. The patient subsequently re-presented with altered mental status and fever. A systolic murmur appreciated at the lower left sternal border, combined with his history of bacteremia, prompted a transthoracic echocardiogram (TTE), which revealed a calcified mobile density suggestive of TV endocarditis (Figure 1). Surgical repair was not deemed feasible due to his comorbidities, and he was treated with a six-week course of meropenem. The patient presented a third time with fever, tachycardia, and hypotension. He underwent a tricuspid valve debulking procedure aimed at reducing the size of the vegetation. Meropenem was continued, but due to an allergic reaction presenting as a rash, his antibiotics were switched to ceftolozane and tazobactam. Despite this change, he was again admitted with worsening chronic decubitus ulcers. The patient developed septic shock secondary to recurrent *P. aeruginosa* bacteremia and was admitted to the ICU, requiring fluid resuscitation and vasopressors. Unfortunately, despite aggressive treatment, he transitioned to comfort care and passed away.

## Discussion

This case underscores the challenge of managing recurrent antibiotic-resistant *P. aeruginosa* infections. The patient’s clinical course was complicated by his paraplegia, chronic ulcerative lesions, indwelling suprapubic catheter, and PPM. Due to his comorbid conditions and a risk-benefit analysis, he was not considered a surgical candidate for TV replacement. The recurrent nature of the bacteremia, despite therapeutic interventions including broad-spectrum antibiotics, early removal of potential sources of re-infection, and a debulking procedure, highlights the risk of bacterial seeding of indolent medical devices when *P. aeruginosa* biofilm formation is suspected. Mechanisms protecting against antibiotics and the host immune response, conferred by the suspected presence of *P. aeruginosa* biofilms on the suprapubic catheter, PPM, and TV, likely contributed to the recurrent nature of his clinical course. Many studies have emphasized this risk, underscoring the need for continued vigilance and innovative strategies to prevent and manage such infections [3]. One such innovation is multifunctional antibacterial-coated devices with local effects, including anti-adhesion, bactericidal, and anti-quorum sensing properties, which have been shown to reduce biofilm formation by *P. aeruginosa *[4]. This patient’s clinical picture was further complicated by his chronic severe decubitus ulcers, which were another possible source of infection [5]. Suppressive antibiotic therapy was considered; however, after antimicrobial susceptibility testing, the patient’s *P. aeruginosa* isolate was found to be resistant to most oral options, including fluoroquinolones [6]. Additional antibiotic options, including aminoglycosides and previously trialed antibiotics, were declined by the patient after discussing the associated risks.

## Conclusions

This case highlights the difficulty of treating recurrent *P. aeruginosa* infections, particularly in the face of growing antibiotic resistance. The patient’s case was further complicated by his indwelling devices and chronic sacral ulcers, which served as potential sources of recurrent infection. The association between implantable medical devices and recurrent bacteremia is well-documented, emphasizing the role of biofilm formation in bacterial colonization, migration, dissemination, and recurrence. Future research should focus on critical areas such as the development of novel antibiotics, biofilm-disrupting agents, antibacterial-coated medical devices, and advanced diagnostic tools to better identify the site of infection. Although the patient was ultimately deemed incurable, this case may inform clinical decision-making by underscoring the importance of addressing each potential source of infection recurrence, including the implementation of novel therapeutic strategies such as coated medical devices. Additionally, this case underscores the broader issue of rising nosocomial infections and the need for antimicrobial stewardship programs to combat the growing threat of antibiotic resistance.
